# Physiognomy Illustrated

**Published:** 1887-07

**Authors:** 


### PHYSIOGNOMY ILLUSTRATED.

The above is the title of a highly interesting book of more than six hundred pages, muslin bound, illustrated by three hundred engravings, by Dr. Joseph Simms, just issued in the eighth edition.

The system of physiognomy formulated by the author, makes no division of the facial, cranial or bodily parts where nature has made none, but it is far more wisely founded upon the anatomical structure of the entire man.

In this new and rational system of reading character, the traits are readily indicated by the Physical *Form*. The Osseous or Bony Form, when it predominates, gives honesty, firmness, observation and a philosophical cast of mind ; small bones show less strength of character, both mental and physical. The Muscular Form indicates activity in trade or traffic, love of music, dancing and the social pleasures of life, and when combined with other physical powers far outstripping the intellectual, they develope into a John L. Sullivan. The Abdominal Form lives to eat, drink and sleep. The Thoracic Form, on the contrary, is ever astir, easily elated, fond of admiration and should never marry one of the same type. The Brain and Nerve Form is shown by the large head, small neck and chest, sharp features, thin lips and nostrils : such characters are keenly sensitive, apt to be dyspeptic, fidgety and highly susceptible to the influence of stimulants. This form gives a thirst for literary fame, and in this thinking age, when very large, it often leads to mental disorders and premature decline.

Eyes that are large and open widely, indicate a disposition to love *one only* for a lifetime ; while eyes that open so little as to have the almond shape, denote the polygamously inclined, as are Arabs, Turks and Chinese. We here learn that the long noses are cautious, while short ones are imprudent, as witnessed in children.

The round, small, well-fluted, thin ear which stands well out from the head, is musical, while large, angular, thick ears are musicless, as seen in the pig and donkey. Large mouths with protrusive flexible lips are naturally oratorical, and when educated properly they become a Demosthenese, Cicero, Webster, Gough or Gambetta. The long, bony face is the benevolent cast of mind, and is willing to work for others like the horse, while the short, wide face like the cat, tiger and puma, are malignant and selfish.

The walk is treated in one chapter, most exhaustively and instructively. The firm gait is found only in strong characters. The long, brisk step accompanies a vigorous, active mind. Laughter is described as an emotion which is confined to the human species, and to laugh well, is the mark of a cultivated gentleman, while a natural laugh cannot be intrinsically unrefined.

The hair forms an instructive chapter, so does also the varieties of shaking hands. One-sided persons are unbalanced mentally, and a host of other signs of character are given on scientific principles, in this natural system of physiognomy, which has never before been presented by any previous author. The writer knows that Dr. Simms cannot be excelled as a practical delineator or reader of personal traits and qualities of mind and body, and this, too, without touching the person examined.

Dr. Bonditch and other gentlemen of the Harvard Medical School, have lately, by scientific experiments, proved that the sense of sight is two hundred and fifty-one times as accurate as that of touch. Now, as physiognomy depends wholly on sight, and phrenology on touch, any one may readily see their relative value and accuracy.

Dr. Simms has abandoned the lecture field after thirty years of unvarying success, and is now travelling in Europe, but persons who desire to obtain the advantages of his vast experience as a skillful and world renowned physiognomist, can procure a copy of this highly instructive work, by sending two dollars in post office order to the Murray Hill Publishing Co., No. 129 East 28th St., New York.

